# Deciphering the Structural Basis of High Thermostability of Dehalogenase from Psychrophilic Bacterium *Marinobacter* sp. ELB17

**DOI:** 10.3390/microorganisms7110498

**Published:** 2019-10-28

**Authors:** Lukas Chrast, Katsiaryna Tratsiak, Joan Planas-Iglesias, Lukas Daniel, Tatyana Prudnikova, Jan Brezovsky, David Bednar, Ivana Kuta Smatanova, Radka Chaloupkova, Jiri Damborsky

**Affiliations:** 1 Loschmidt Laboratories, Department of Experimental Biology and RECETOX, Faculty of Science, Masaryk University, Kamenice 5/A13, 625 00 Brno, Czech Republic; lukchrast@gmail.com (L.C.); 211165@mail.muni.cz (L.D.); janbre@amu.edu.pl (J.B.); radka@chemi.muni.cz (R.C.); 2 Institute of Chemistry and Biochemistry, Faculty of Science, University of South Bohemia Ceske Budejovice and Institute of Microbiology Academy of Sciences of the Czech Republic, Branisovska 1760, 370 05 Ceske Budejovice, Czech Republic; ktratsiak@gmail.com (K.T.); talianensis@gmail.com (T.P.); ivanaks@seznam.cz (I.K.S.); 3 Institute of Organic Chemistry and Biochemistry, Czech Academy of Sciences, v.v.i., Flemingovo nam. 2, 166 10 Prague 6, Czech Republic; 4 International Clinical Research Center, St. Anne’s University Hospital, Pekarska 53, 656 91 Brno, Czech Republic; 5 Enantis Ltd., Biotechnology Incubator INBIT, Kamenice 771/34, 625 00 Brno, Czech Republic

**Keywords:** haloalkane dehalogenase, thermostability, psychrophile, access tunnel, dimer, catalytic pentad, enantiselectivity

## Abstract

Haloalkane dehalogenases are enzymes with a broad application potential in biocatalysis, bioremediation, biosensing and cell imaging. The new haloalkane dehalogenase DmxA originating from the psychrophilic bacterium *Marinobacter* sp. ELB17 surprisingly possesses the highest thermal stability (apparent melting temperature *T*_m,app_ = 65.9 °C) of all biochemically characterized wild type haloalkane dehalogenases belonging to subfamily II. The enzyme was successfully expressed and its crystal structure was solved at 1.45 Å resolution. DmxA structure contains several features distinct from known members of haloalkane dehalogenase family: (i) a unique composition of catalytic residues; (ii) a dimeric state mediated by a disulfide bridge; and (iii) narrow tunnels connecting the enzyme active site with the surrounding solvent. The importance of narrow tunnels in such paradoxically high stability of DmxA enzyme was confirmed by computational protein design and mutagenesis experiments.

## 1. Introduction

Haloalkane dehalogenases (HLDs; EC 3.8.1.5) form a family of enzymes that catalyze hydrolytic cleavage of carbon-halogen bond in a wide range of halogenated aliphatic hydrocarbons and their derivatives via S_N_2 nucleophilic substitution followed by the addition of water, and releasing a halide ion, one proton, and the corresponding alcohol as reaction products [1,2]. Apart from the molecule of water required for catalytic activity, these enzymes do not demand any other cofactors. Structurally, HLDs belong to the α/β-hydrolases superfamily, a well-distinguished group of structurally similar enzymes containing peptidases, esterases, lipases or epoxide hydrolases [3].

The structure of HLDs is composed of a conserved main (or core) domain, consisting of eight-stranded β-sheet and six α-helices, and a versatile cap domain formed mostly by α-helices. The active site contains a catalytic pentad and is placed in a hydrophobic pocket buried between the main and the cap domain. The active site of HLDs is accessible via the main and slot tunnels [4,5], both of them being important determinants of enzyme activity and substrate specificity [6,7]. The catalytic pentad of HLDs consists of a nucleophile, a base, a catalytic acid, and two halide-stabilizing residues [1]. The composition of the catalytic pentad varies among the three HLD subfamilies identified by phylogenetic analysis: Asp-His-Asp+Trp-Trp in HLD-I, Asp-His-Glu+Asn-Trp/Tyr in HLD-II, and Asp-His-Asp+Asn-Trp in HLD-III [8,9].

It has been found that over 200 halogenated compounds are substrates for HLDs including brominated, chlorinated, and iodinated compounds [10]. Based on the preferences of each HLD member towards particular halogenated substrates, the enzymes can be divided into four substrate specificity groups [10], which do not correspond with phylogenetic subfamilies. It is impossible thus far to predict substrate specificity of the putative HLDs because of different structural features affecting the enzyme activity [8,10].

HLDs can be found in many microorganisms inhabiting soil and marine environments, some of them were also identified in pathogenic mycobacterial strains. HLDs hold great potential in several practical applications including biodegradation, biocatalysis, biosensing, neutralization of chemical warfare agents, and cell imaging, as reviewed by Koudelakova et al. [11] and Nagata et al. [12].

Organisms inhabiting extreme environments represent a valuable source of enzymes with unique properties highly important for biocatalysis and other biotechnological applications [13,14]. Consequently, thermostable enzymes have attracted most attention among all extremozymes during the past decade. Among other structural features of thermostable and thermotolerant enzymes, the inner hydrophobicity and compactness seem to be a factor of paramount importance for preserving their structure at elevated temperatures [15]. Thermophilic enzymes are well known for their reduced flexibility [16] that sporadically is merely reduced to a local rigidity of the protein structure, which alone might be sufficient for maintaining the structural integrity at high temperatures [17]. The enhanced stability of thermophilic proteins can be further attributed to a higher proportion of charged and hydrophobic residues [18], the presence of salt bridges [19] and multiple electrostatic interactions [18], or an increased proportion of hydrogen bonds on the main chain of such enzymes [15,20].

Interestingly, some thermostable enzymes were also isolated from psychrophilic and mesophilic organisms, e.g., aldehyde dehydrogenase [21], aspartase [22] and alcohol dehydrogenase [23] from *Flavobacterium frigidimaris* KUC-1; endoglucanase from *Fusarium oxysporum* L19 [24]; isocitrate dehydrogenase from *Desulfotalea psychrophila* LSv54 [25]; or haloacid dehalogenase from *Psychromonas ingrahamii* DSMZ 17664 [26]. However, a paradigm of thermostable enzymes from psychrophiles has not yet been conclusively explained and it is not known why cold-adapted organisms possess highly stable enzymes. Novak and colleagues suggested the important role of horizontal gene transfer as the reason for the presence of the thermostable enzyme in the psychrophilic organism [26].

In the present paper, we describe the biochemical and structural characterization of a new HLD from psychrophilic bacterium *Marinobacter* sp. ELB17, paradoxically possessing the highest thermostability among all biochemically characterized wild type enzymes from HLD-II subfamily. Crystallographic analysis revealed three unique features of DmxA that can contribute to its unusual thermostability: (i) a unique composition of catalytic pentad, possibly lacking one out of the two halide-stabilizing residues; (ii) enzyme dimerization due to a disulfide bridge; and (iii) unusually narrow tunnels connecting the buried enzyme active site with the surrounding solvent. We carefully investigated all unique features of DmxA in order to explain their role in the enzyme stability. Molecular dynamic simulations followed by mutagenesis and functional characterization of DmxA variants revealed a shift in substrate specificity and dramatically modified thermostability, thereby providing insight into the role of access tunnels for the paradoxical robustness of this enzyme.

## 2. Materials and Methods

### 2.1. Gene Synthesis, Expression, and Purification

The *dmxA* gene with sequence from GeneBank database (whole genome shotgun sequence accession number AAXY01000003.1, nucleotides 263,779–264,669; genomic DNA translation accession number EBA00776.1) was artificially synthesized (Mr.Gene, Regensburg, Germany; newly Thermo Fisher Scientific, Waltham, Massachusetts, USA). The codon usage was adapted to the codon bias of *E. coli* genes by Mr. Gene’s website service. For expression purposes, the gene containing His-tag was subcloned to the expression vector pET21b (Novagen, Madison, Wisconsin, USA) between restriction sites *Nde*I and *Hind*III.

Heterologous expression of DmxA wt and all variants was performed in LB medium (Sigma-Aldrich, St. Louis, Missouri USA). The medium was composed of 10 g/L tryptone, 5 g/L yeast extract, and 5 g/L sodium chloride [27]. Precultures were prepared by picking one colony of transformed *Escherichia coli* carrying the gene coding a target enzyme to 10 mL of LB medium with ampicillin and incubated overnight at 37 °C and 200 rpm. One liter of LB medium supplemented with ampicillin was inoculated with the overnight culture and incubated at 37 °C and 120 rpm. When the culture reached OD_600_ 0.5, the expression was initiated by the addition of isopropyl β-D-1-thiogalactopyranoside to a final concentration of 0.5 mM and cells were further incubated overnight at 25 °C. Cells were harvested by centrifugation at 6000 *g* and 4 °C, stored at - 70 °C and defrosted before purification.

Harvested cells were disrupted by sonication using a Soniprep 150 (Sanyo Gallenkamp PLC, Loughborough, UK). The supernatants were collected after centrifugation at 21,000 g for 1 h. Cell-free extract was applied on 5 mL Ni-NTA Superflow column charged by Ni^2+^ ions (Qiagen, Hilden, Germany) in equilibrating buffer (20 mM potassium phosphate buffer, pH 7.5, containing 0.5 M sodium chloride, and 10 mM imidazole). Unbound and weakly bound proteins were washed out with the buffer containing 10 mM imidazole. The target enzyme was eluted with a buffer containing 300 mM imidazole. The active fractions were pooled and dialyzed against 50 mM potassium phosphate buffer, pH 7.5, overnight. DmxA and its variants, all containing a C-terminal hexahistidyl tail, were stored at 4 °C in 50 mM potassium phosphate buffer prior to analysis. Expression profiles, solubility, and purity of the enzymes were checked by SDS-PAGE; the amount of target enzyme in the fractions on SDS gel was determined by a GS-800 Calibrated Densitometer (Bio-Rad, Hercules, California, USA). The concentration of purified enzyme was determined by the Bradford method [28], using bovine serum albumin as a standard.

### 2.2. Analysis of Secondary Structure and Thermostability

Circular dichroism (CD) spectra were recorded at room temperature using a Chirascan spectrometer (Applied Photophysics, Leatherhead, UK) equipped with a Peltier thermostat. Data were collected from 185 to 260 nm, at a scan rate of 100 nm/min, 1 s response time and 1 nm bandwidth using a 0.1 cm quartz cuvette containing 0.2 mg/mL enzyme in a 50 mM potassium phosphate buffer (pH 7.5). Each collected spectrum represents an average of five individual scans and has been corrected for absorbance caused by the buffer. CD data were expressed in terms of the mean residue ellipticity (*Θ*_MRE_) using the following equation:

*Θ*_MRE_ = (*Θ*_obs_⋅*M*_w_⋅100)/(*n*⋅*c*⋅*l*) (1)

where *Θ*_obs_ is the observed ellipticity in degrees, *M*_w_ is the protein molecular weight, *n* is a number of residues, *l* is the cell path length, *c* is the protein concentration, and the factor of 100 originates from the conversion of the molecular weight to mg/dmol.

Thermal unfolding of DmxA wt and its variants was followed by monitoring the ellipticity at 222 nm over a temperature range of 20 to 80 °C, using a resolution of 0.1 °C and the heating rate of 1 °C/min. The resulting thermal denaturation was roughly normalized to represent a signal change between approximately 1 and 0 and fitted to sigmoidal curves using Origin 6.0 software (OriginLab, Northampton, Massachusetts, USA). The apparent melting temperature (*T*_m,app_) was evaluated as a midpoint of the normalized thermal transition.

### 2.3. Native Polyacrylamide Gel Electrophoresis

Disulfide bond formation in DmxA wt was studied by comparing the size of reduced and non-reduced enzyme samples on the native polyacrylamide gel. Two well-known HLDs, LinB (34 kDa, monomer) and DbjA (66 kDa, dimer) were used as molecular weight standards. To obtain the enzyme in reducing conditions, the solution of DmxA was diluted to 4 mg/mL and dithiothreitol (DTT) was added to final concentration 10 mM. The sample was then degassed at room temperature for 30 min and saturated with the 20-fold volume of pure nitrogen. Protein samples were mixed with loading dye and applied to the gel wells. The electrophoresis experiments were performed at 4 °C and 115 V. The gel was stained with Coomassie Brilliant blue R250 (Sigma-Aldrich, St. Louis, Missouri, USA) and analyzed by GS-800 Calibrated Densitometer (Bio-Rad, Hercules, California, USA).

### 2.4. Specific Activity Assay

Enzymatic activity was assayed using the colorimetric method developed by Iwasaki and colleagues [29]. The release of halide ions was measured spectrophotometrically at 460 nm using a SUNRISE microplate reader (Tecan, Salzburg, Austria) after the reaction with mercuric thiocyanate and ferric ammonium sulfate. Dehalogenation reactions were performed at 37 °C in 25-mL Reacti-flasks closed by Mininert valves. The reaction mixture contained 10 mL of glycine buffer (100 mM, pH 8.6) and 10 μL of the halogenated substrate. The reactions were initiated by the addition of appropriate amount of the enzyme depending on its activity. The reactions were monitored by withdrawing 1 mL samples at periodical intervals from the reaction mixture and immediately mixing the samples with 0.1 mL of 35% nitric acid to terminate the reaction. Dehalogenation activities were quantified as rate of product formation with time.

The effect of temperature on activity of DmxA and its variants was determined by performing activity assay at different temperatures. The activity measurements were evaluated at temperatures ranging from 20 to 65 °C in 100 mM glycine buffer pH 8.6. Activity measurements were performed with 1,3-diiodopropane.

### 2.5. Principal Component Analysis (PCA) Analysis

A matrix containing the activity data for ten wild-type HLDs and three DmxA variants with 30 substrates was analyzed by PCA to uncover relationships between individual HLDs (cases) and their substrates (variables) [10]. In brief, PCA was performed using Statistica 12.0 (StatSoft, Tulsa Oklahoma, USA). The raw data were log-transformed and weighted relative to the individual enzyme’s activity towards other substrates prior to analysis, in order to better discern individual enzymes’ specificity profiles. Thus: (i) each specific activity value was incremented by 1 unit; (ii) the log of this new value was taken; and (iii) this log value was then divided by the sum of all the log values for that particular enzyme. The transformed data were used to identify substrate-specificity groups of enzymes that exhibited similar specificity profiles regardless of their overall specific activities.

### 2.6. Freeze–Thaw Stability Testing

To determine cold stability of DmxA wt and its variants, the effect of numerous freeze–thaw cycles on the enzyme activity was followed. The enzyme samples prepared at 1 mg/mL concentration in 50 mM phosphate buffer pH 7.5 were injected in 2 mL glass tubes closed by rubber stopper and aluminum cap. The samples were subsequently frozen at −80 °C. After freezing the particular enzyme sample was put to room temperature and naturally thawed. When fully thawed, an aliquot was withdrawn from the stock solution and the remaining enzyme solution was frozen again, up to 10 freeze–thaw cycles. The cold stability of the enzymes was monitored by determination of enzyme activity before and after each freeze–thaw cycle. The enzyme activity was assayed with 1,3-dibromopropane as substrate in 100 mM glycine buffer pH 8.6 at 37 °C as described above. Each activity was measured in three to five independent replicates and represented as mean values of relative activity with plotted standard deviations. Relative activities represented a percentage of specific activity of the enzyme before freezing.

### 2.7. Steady-State Kinetics

The steady-state kinetics of DmxA wt and its variants were determined with three halogenated substrates by using a VP-ITC isothermal titration calorimeter (MicroCal, Piscataway, New Jersey, USA) at 37 °C. A reaction mixture vessel of the microcalorimeter was filled with 1.4 mL of enzyme solution at a concentration of 0.005–0.06 mg/mL (enzyme was dialyzed against 100 mM glycine buffer, pH 8.6). The substrate solution was prepared in the same buffer by the addition of 1,2-dibromoethane, 1,3-dibromopropane or 4-bromobutyronitrile to a final concentration of 22–28, 20–23, and 6–8 mM, respectively. Substrate concentration was verified by gas chromatography (Agilent Technologies, Santa Clara, California USA) prior to the experiment. In the kinetic experiment, the enzyme was titrated in 150 s intervals in the reaction mixture vessel with increasing amounts of the substrate, while pseudo-first-order conditions were maintained. Every 10 µL injection increased the substrate concentration, leading to a further increase in enzyme reaction rate (an increase of heat generation) until the enzymatic reaction was saturated. In total, 28 injections were performed during titration. The reaction rates reached during every injection (in units of thermal power) were recalculated to enzyme turnover. The calculated enzyme turnover plotted against the actual concentration of the substrate after every injection was then fitted by nonlinear regression to different kinetic models using Origin 6.0 (OriginLab, Northampton, Massachusetts, USA).

### 2.8. Measurement of Enantioselectivity

Enantioselectivity measurement was performed at 20 °C in 25-mL Reacti-flasks closed by Mininert valves containing 25 mL of Tris-sulfate buffer (50 mM, pH 8.2). The racemic substrates (2-bromopentane and ethyl 2-bromopropionate) were added to the reaction mixture to a final concentration of 0.5–3 mM. The reaction was initiated by addition of 1 mL of an enzyme (5–7 mg/mL) into the reaction mixture. The reaction progress was monitored by periodical withdrawing samples from the reaction mixture. The samples were mixed with methanol containing 1,2-dichloroethane as an internal standard. The samples were analyzed by using Agilent Technologies 7890A gas chromatograph (Agilent Technologies, Santa Clara, California, USA) equipped with a flame ionization detector and chiral capillary column Astec Chiraldex B-DM (50 m × 0.25 mm × 0.12 μL film thickness) (Sigma-Aldrich, St. Louis, Missouri, USA). The enantioselectivity was expressed as *E*-value defined as the ratio between the specificity constants (*k*_cat_/*K*_m_) for the two enantiomers. To estimate the kinetic parameters, the equation describing competitive Michaelis–Menten kinetics was fitted by numerical integration to progress curves obtained from the kinetic resolution experiments by using software Scientist (MicroMath Research, St. Louis, Missouri, USA).

### 2.9. Enzyme Crystallization

The purified DmxA enzyme in a 50 mM Tris-HCl buffer (pH 7.5) at the concentration of 10 mg/mL was used for the crystallization experiments. The crystallization procedure was performed as described previously by Tratsiak et al. [30] with small variation: sitting crystallization drop consisted of 12 µL of the protein solution, 6 µL of precipitant solution, and 3.6 µL of 0.1 M sarcosine (Hampton Research, Aliso Viejo, California, USA) was equilibrated against 2 mL of a reservoir solution in the crystallization mushroom (Triana Science & Technology, Armilla, Spain). The rhombohedral-shaped crystals with the dimensions of approximately 160 µm × 100 µm × 150 µm appeared after 9 days of incubation at 20 °C.

### 2.10. Data Collection and Processing

Single crystals of DmxA were mounted into MicroLoops (MiTeGen; Jena Bioscience GmbH, Jena, Germany) and directly flash-cooled in a liquid-nitrogen stream without additional cryoprotection. The diffraction data were collected at beamline ID29 at the ESRF (European Synchrotron Radiation Facility, Grenoble, France) [31] at the wavelength of 0.972 Å and −173 °C using a Pilatus 6M-F detector (DECTRIS Ltd., Baden, Switzerland). A complete diffraction dataset of 3000 images with 0.05° oscillation and 265 mm crystal-to-detector distance was collected up to 1.45 Å resolution. The diffraction data were processed by XDS program package [32,33,34]. Crystal parameters and data collection statistics are summarized in Table 1.

### 2.11. Structure Determination and Refinement

The phase problem was solved by the molecular replacement method using MOLREP [35] with the structure of DhaA from *Rhodococcus* sp. (PDB ID 4E46) [36] as a search model. The structure was refined by restrained isotropic and TLS refinement [37,38,39,40] using 2 TLS groups and local NCS refinement with 1 NCS group carried out by REFMAC5 (version 8.0158) [41] from the CCP4 (version 7.0.035) package [39]. Manual building steps were performed in COOT [42]. The last steps of the structure refinement were also carried out by the web server PDB_REDO [43] for structure model optimization.

The structure validation and analyses were performed using MOLPROBITY service [44], SFCHECK [45], wwPDB Validation Server [46], LSQKAB [47], and PISA server [48]. Atomic coordinates and experimental structure factors were deposited in the RCSB Protein Data Bank under PDB code 5MXP.

### 2.12. Molecular Dynamics

Interactions between residues and their influence on DmxA performance were studied by molecular dynamics (MD) simulations, using dimeric crystal structure data. The hydrogen atoms were added to both DmxA units separately with H++ server at pH 7.5 [49]. All water molecules from the crystal structure were added to the systems. Cl^−^ and Na^+^ ions were added to the final concentration of 0.1 M using Tleap module of AMBER 14. An octahedron of TIP3P water molecules [50] was also added to the distance of 10 Å from any atom in the system using the same module in the case of the short-run wt and mutant systems or the solvate method from HTMD [51] in the case of the long-run wt systems.

The short-run and mutant systems were minimized in five rounds consisting of 5000 steepest descent steps followed by 5000 conjugate gradient steps with a decreasing restraint on the protein backbone (500, 125, 50, 25 and 0 kcal mol^−1^ Å^−2^). The following MD simulations employed periodic boundary conditions, the particle mesh Ewald method for treatment of the electrostatic interactions [52], 10 Å cutoff for nonbonded interactions, and 2 ft time step with the SHAKE algorithm [53] to fix all bonds containing hydrogens. The equilibration simulation consisted of two steps: (i) 20 ps of gradual heating from 0 to 310 K under constant volume, using a Langevin thermostat with collision frequency of 1.0 ps^−1^, and with harmonic restraints of 5.0 kcal mol^−1^ Å^−2^ on the position of all protein atoms; and (ii) 2000 ps of unrestrained MD at 310 K using the Langevin thermostat, and constant pressure of 1.0 bar using pressure coupling constant of 1.0 ps^−1^. Finally, production MD simulations were run for 150 ns with the same settings as the second step of equilibration MD. Coordinates were saved in 2 ps interval, and the trajectories were analyzed using cpptraj module [54] from AMBER 14. All calculations were carried out in the GPU (CUDA) PMEMD module [55,56] of AMBER 14 using ff14SB force field [57,58,59].

The long-run systems were equilibrated starting with a 500-step conjugate gradient minimization, followed by heating to 300 K using the equilibration protocol of HTMD as follows: (i) 2.5 ns of NPT equilibration with Langevin thermostat with 1 kcal·mol^−1^·Å^−2^ with constraints of all heavy atoms of the protein followed by (ii) 2.5 ns of equilibration under the same conditions but no constraints. Holonomic constraints were placed on all hydrogen-heavy atom bond terms and the mass of hydrogen atoms was scaled with factor 3 enabling a 4 fs time-step [60,61,62,63]. The production simulations were run as adaptive epochs at 300 K using the same settings as in the last step of the equilibration. The metric used in the adaptive sampling was the distances of Cα atoms in the structure. The simulations were run for 50 epochs of 10 × 100 ns (total 50 μs of simulation time) and the frames were saved every 0.1 ns. All trajectories for the wt and the mutant systems were visualized in PyMOL [64] and VMD 1.9.1 [65].

### 2.13. Tunnel Analysis

Tunnel networks were analyzed by the stand-alone version of CAVER 3.02 [66]. Each atom of the protein structure was approximated by 12 spheres. The starting point was specified by residues Asp 105, Trp 106, Asn 40 and Phe 169 followed by automatic optimization to prevent collision with other protein atoms. The tunnel search was performed on every second frame from the MD by using probe radius of 0.8 Å and the default settings. The redundant tunnels were automatically removed from each snapshot. The clustering of tunnels was performed by the average-link hierarchical Murtagh algorithm based on the calculated matrix of pairwise tunnel distances. The clustering threshold was set to 4.

### 2.14. Construction of Mutants

All mutants were designed by Rosetta 3.3. The structures of both monomers DmxA were first minimized by a Rosetta routine minimize_with_cst with the following parameters: both backbone and side chains optimization was enabled (sc_min_only false), distance for full atom pair potential was set to 9 Å (fa_max_dis 9.0), and standard weights for the individual terms in the energy function were used and constraint weight 1 (constraint_weight 1.0). The output from minimization was used by script convert_to_cst_file.sh for a creation of the constraint file. The construction of mutants was performed by the ddg_monomer module of Rosetta by using parameters employed in protocol 16 by Kellogg et al. [67]. The soft-repulsive design energy function (soft_rep_design weights) was used for side chains repacking and backbone minimization (sc_min_only false). Optimization was performed on whole protein without distance restriction (local_opt_only false). Previously created cst file was used as a constraint during backbone minimization (min_cst true). The optimization was performed in three rounds with increasing weight on the repulsive term (ramp_repulsive true). A minimum energy (mean false, min true) from 50 iterations (iterations 50) was employed as a final parameter describing the stability effect.

### 2.15. Site-Directed Mutagenesis

Primers for the preparation of genes encoding variants of DmxA were designed by using CloneManager 10 (Sci-Ed software, Cary, North-Carolina, USA). Sequences of mutagenic and non-mutagenic primers are available in Table S6. The site-directed mutagenesis was performed according to modified protocol described by Sanchis et al. [68]. PCR mixtures were prepared by mixing 100 ng of the template plasmid pET21b::re_*dmxA*, 1 µL of each 10 mM primer, 1 µL of 10 mM mixture of dNTPs, 1.25 µL of Phusion High fidelity polymerase (2.5 U), 10 µL of 5× Phusion High fidelity polymerase buffer, and sterile water to final volume 50 µL. In the first part of PCR, the megaprimers were formed between mutagenic primers or mutagenic and non-mutagenic primers. In the second part of PCR, the megaprimers served as primers for replication of the whole plasmid.

Template DNA was digested with *Dpn*I endonuclease during 1 h incubation at 37 °C. The endonuclease in the mixture was deactivated by incubation at 80 °C for 10 min. Then, 5 µL of the PCR mixture was digested by *Nde*I and *Hin*dIII restriction endonucleases and analyzed by agarose gel electrophoresis; 5–7 µL of the mixture were transformed to *E. coli* DH5α cells. After overnight cultivation at 37 °C, 4–6 colonies were picked from the agar plates and used for the preparation of 10-mL overnight cultures. Plasmids were isolated from cultured cells using GeneJET Plasmid Miniprep Kit (Fermentas, Burlington, Canada) and sequenced on both strands. Simultaneously, glycerol stocks were prepared from the overnight cultures by mixing with 30% glycerol (1:1).

## 3. Results

### 3.1. Expression and Biochemical Characterization of DmxA

**Figure 1:**
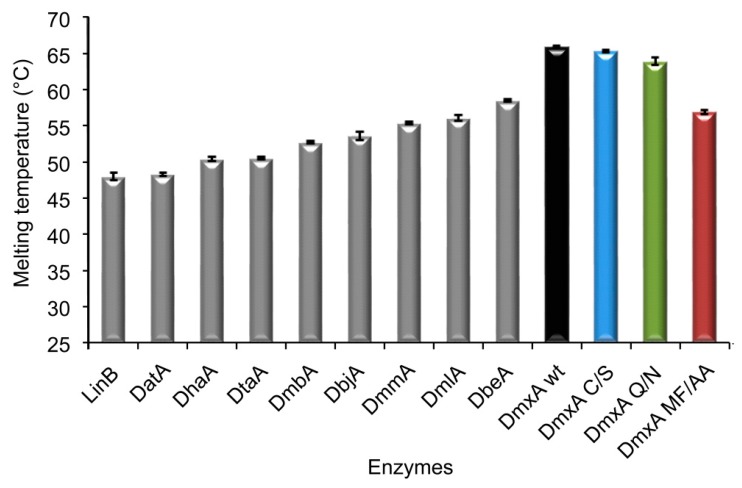
Comparison of melting temperatures of selected biochemically characterized HLD-II members with haloalkane dehalogenase DmxA wt and its variants constructed in this study. Wild type DmxA exhibits the highest stability (*T*_m,app_ = 65.9 ± 0.1 °C) of so far characterized enzymes from HLD-II subfamily. Error bars represent standard deviations from at least three independent experiments.

The gene encoding putative haloalkane dehalogenase DmxA was identified in the genome of *Marinobacter* sp. ELB17, a psychrophilic gammaproteobacterium isolated from the east lobe of Lake Bonney in the Taylor Valley, Antarctica [69]. The artificially synthesized *dmxA* gene (Figure S1) was subcloned into expression vector pET21b, overexpressed in *E. coli* BL21(DE3), and the His-tagged protein was purified to homogeneity with an average yield of 390 mg of soluble protein per liter of cell culture and purity over 95%. The folding, secondary structure, and stability of the enzyme were analyzed by CD spectroscopy. Similar to other related HLDs, DmxA wt exhibited CD spectrum with one positive peak at 195 nm and two negative maxima at 208 and 222 nm (Figure S2), characteristic of α-helical content [70], thus suggesting a proper folding of the enzyme. Thermally induced denaturation experiments revealed that DmxA wt exhibits the highest apparent melting temperature (*T*_m,app_ = 65.9 ± 0.1 °C) determined thus far for wild-type enzymes belonging to HLD-II (Figure 1). Similar to other members of haloalkane dehalogenase family, the thermal unfolding of DmxA wt is irreversible (data not shown). However, the reported stability of the enzyme is paradoxical considering that its source organism, *Marinobacter* sp. ELB17, grows optimally at 12–15 °C [69]. The oligomeric state of DmxA wt was determined by gel filtration chromatography and native PAGE electrophoresis (Figures S3 and S4). DmxA wt was found to exist in a solution in monomer-dimer equilibrium under tested conditions.

**Figure 2:**
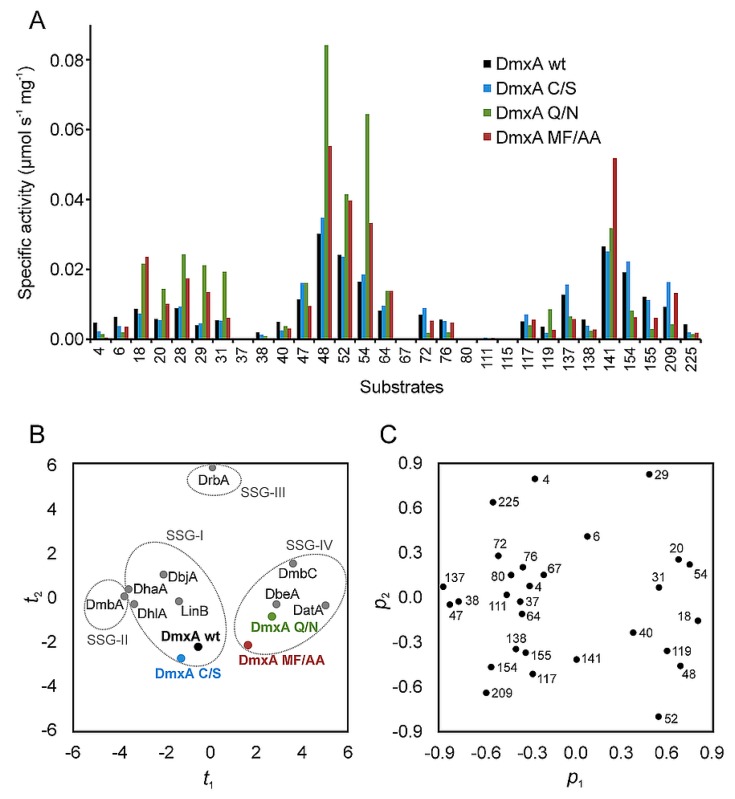
Substrate specificity of DmxA wt and its variants. (**A**) The substrate specificity profiles determined with thirty halogenated substrates. DmxA wt exhibits activity toward 26 out of 30 substrates. (**B**) The score plot *t*_1_/*t*_2_ from principal component analysis with a transformed dataset. The score plot is a two-dimensional window into the multidimensional space, where the objects (enzymes) with similar properties (specificity profiles) are collocated. The *t*_1_/*t*_2_ score plot describing 44% of variance in the dataset shows clustering of HLDs into individual substrate specificity groups (SSGs). Unlike DmxA wt and DmxA C/S, both belonging to SSG-I, DmxA Q/N and DmxA MF/AA were clustered into SSG-IV together with DbeA, DatA and DmbC. (**C**) The corresponding loading plot *p*_1_/*p*_2_ from the principal component analysis showing the substrates that govern the clustering of enzymes into individual SSGs. Variables localized further from the origin contribute to the principal component more than the variables localized closer to the origin of the plot. The numbering of the substrates is provided in Table S1.

The substrate specificity of DmxA wt was assayed towards a set of 30 different halogenated substrates, representing a wide range of chemical structures with varied physiochemical properties [10]. DmxA wt exhibited activity towards 26 of 30 tested compounds (Figure 2A) preferring brominated and bromo-chlorinated substrates to chlorinated substrates, which elicited a poor enzymatic activity (Table S1). The highest enzyme activity (0.030 μmol s^−1^ mg^−1^) was determined with 1,3-dibromopropane. Although the overall activity of the enzyme is relatively low compared to other HLDs, PCA classified DmxA wt into the substrate-specificity group I (SSG-I) together with DbjA, DhaA, DhlA, and LinB (Figure 2B). All enzymes belonging to SSG-I possess broad substrate specificity and usually exhibit activity towards most of the tested substrates including poorly degradable chlorinated compounds [10].

The catalytic properties of DmxA wt were assessed by measuring steady-state kinetics with 1,2-dibromoethane, 1,3-dibromopropane and 4-bromobutyronitrile (Table S2). The most complex kinetic mechanism of DmxA wt was observed in the reaction with 1,3-dibromopropane, involving cooperativity and partial substrate inhibition. DmxA wt kinetics with 1,2-dibromoethane and 4-bromobutyronitrile follow a mechanism involving substrate inhibition. The strongest substrate inhibition of DmxA wt was observed on the 1,2-dibromoethane reaction, explaining the relatively low activity of the enzyme towards this substrate. On the contrary, the highest catalytic efficiency of the enzyme was detected in the reaction with 1,3-dibromopropane, for which DmxA wt exhibited approximately 20-times lower *K*_m_ than for other two tested substrates, corroborating the highest activity of the enzyme towards the substrate.

The enantioselectivity of DmxA wt was assessed by determining the kinetic resolution of racemic 2-bromopentane and ethyl 2-bromopropionate. Similar to other HLDs, DmxA exhibited excellent enantioselectivity towards the tested α-brominated ester (*E*-value > 200) and also high enantioselectivity towards the β-brominated alkane (*E*-value = 100), with a preference for the (*R*)- over the (*S*)-enantiomer for both compounds (Table S3). The high enantioselectivity towards 2-bromopentane (*E*-value > 100) was previously observed for DbjA [71] and DatA [9], while other HLDs exhibited low enantioselectivity towards this substrate [71].

### 3.2. Structural Characterization of DmxA

**Figure 3:**
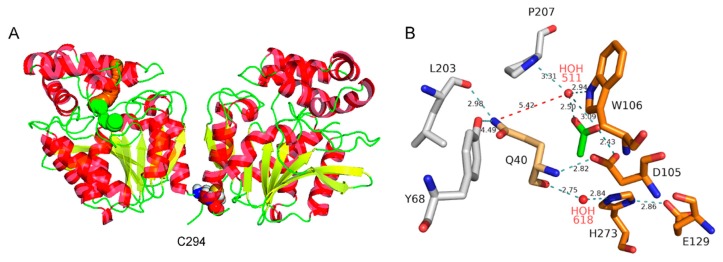
Structural analysis of DmxA. (**A**) The overall structure of DmxA homodimer shown in cartoon representation. The β-sheets are shown in yellow; the α-helices in red; the C294 residue forming an intramolecular bridge is highlighted by spheres; the main and the slot tunnels connecting the active site with the enzyme surface are shown as orange and green spheres, respectively. (**B**) Detail of the DmxA active site. Catalytic residues, acetate ion and residues interacting with Q40 displayed in stick representation are shown in orange, green and grey color, respectively. The water molecules are displayed as spheres. Hydrogen bonds are represented by dashed lines.

The crystal structure of DmxA wt (Figure 3) was determined by molecular replacement and refined using diffraction data to 1.45 Å (Table 1). DmxA wt was crystallized in a primitive orthorhombic crystal form, encompassing two protein molecules in the asymmetric unit. The two molecules in the asymmetric unit represent the dimeric form of the enzyme, which was also observed in solution (Figures S3 and S4). The two N-terminal residues could not be modeled into the electron density map and are thus missing in both molecules in the asymmetric unit. In addition, the C-terminal His tag sequence, representing a cloning artifact, was not modeled. The RMSD for the superposition of 288 Cα atoms of the two protein chains in the asymmetric unit was 0.984 Å. Residues 139–145 and the C-terminal region encompassing residues 294–296 show higher differences, with an RMSD over 1.5 Å. These regions are located on the protein surface and the structural differences reflect their inherent flexibility and participation in different crystal contacts. Chain A is herein used for further description.

The overall structure and domain organization of DmxA wt is very similar to other related HLDs (Figure 3A). The closest structural homolog of DmxA wt is DhaA from *Rhodococcus rhodochrous* (PDB ID 4E46) [36], 292 residues of which were superimposed to DmxA using DALI [72] with an RMSD of 1.1 Å. The core domain comprises typical α/β-hydrolase structure consisting of eight-stranded β-sheet (with β2 lying in an antiparallel orientation) flanked by six α-helices. The cap domain, inserted between β-strand β6 and α-helix α8, consists of five short α-helices connected by six loop insertions. The active site is located in a predominantly hydrophobic cavity, at the interface between the core and the cap domains, connected to the protein surface by two access tunnels.

DmxA wt contains four out of the five canonical catalytic residues in the active site: the nucleophile D105, the catalytic acid E129 and the catalytic base H273, forming catalytic triad, and one halide-stabilizing residue W106 (Figure 3B). The second halide-stabilizing residue, which is asparagine in HLD-II subfamily, is Q40 in DmxA wt.

During the refinement of DmxA wt crystal structure, the electron density present in the vicinity of the active site was interpreted as a water molecule and an acetate ion with an occupancy of 1.0 for both molecules present in the asymmetric unit. The water molecule (HOH511) is situated in a canonical halide-binding pocket of the enzyme (Figure 3B) and interacts with the nitrogen atom of one halide-stabilizing residue W106 N^ε^1 at a distance of 2.94 Å. Further coordination of the water molecule was provided by two oxygen atoms from the ketone and the hydroxyl group of the acetate molecule at 2.50 Å and 3.09 Å, respectively, and the nitrogen from P207 at 3.31 Å (Figure 3B). The residue Q40 is not involved in contact with HOH511, Q40 N^ε^2 is situated 5.42 Å far from the water molecule. The side chain of Q40 faces away from the halide-binding site and makes polar interactions with the side chain hydroxyl of Y68 and the main chain carbonyl of L203. This suggests that Q40 residue cannot be involved in the stabilization of the substrate nor releasing halide anion in DmxA wt active site. The catalytic nucleophile D105 further interacted with the acetate anion by the formation of hydrogen bonds between D105 O^δ^1 and oxygen atom O from the hydroxyl group of the acetate ion at 2.43 Å.

The analysis of intermolecular contacts in the structure suggested that DmxA wt dimerization is mediated through the interaction of the C-terminal helix (residues 281–296) and the β8 sheet (residues 263–267) of one monomer with the corresponding secondary structure elements of the other one. Twenty-one residues are involved in the formation of the dimer interface. Buried solvent-accessible area is 610.6 Å^2^, representing 5.3% of the total solvent accessible surface area of a monomer. Interactions in the dimeric interface are mediated by 3 salt bridges, 10 hydrogen bonds and, interestingly, an intermolecular disulfide bond formed by cysteine 294 residues from each monomer. DmxA wt represents the first example of oligomerization via disulfide bridge formation reported in the HLD enzyme family thus far.

### 3.3. Construction of Variant DmxA C/S with Eliminated Cysteine Bridge

**Figure 4:**
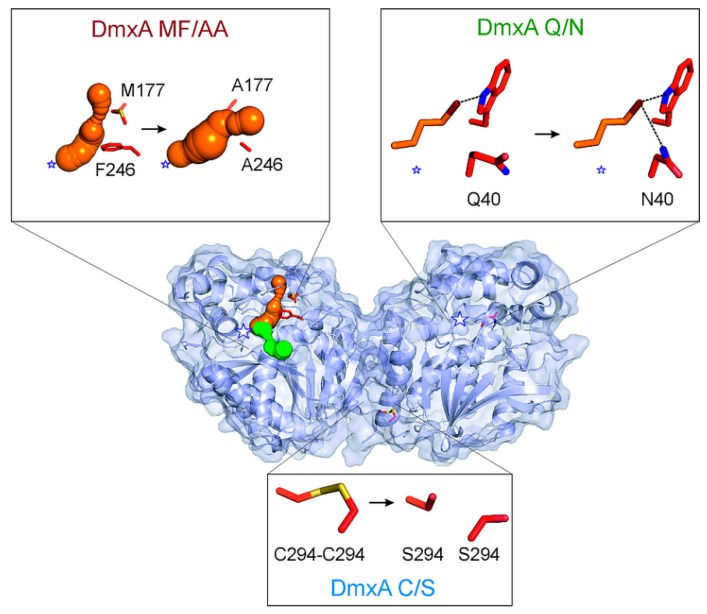
The overall structure of DmxA dimer represented by a diagram showing the solvent accessible surface. The main tunnel connecting the surface and the surrounding solvent with the hydrophobic pocket containing the catalytic pentad is shown in orange and residues forming the tunnel bottleneck are highlighted in sticks. The slot tunnel accessing the active site of DmxA wt is depicted as green sphere. DmxA wt is the only dehalogenase forming a dimer containing disulfide bridge between C294 residues. Mutant DmxA C/S was constructed by replacing C294 with serine to examine the effect of cysteine bridge on stability and dimerization of DmxA. The non-contributing residue Q40 is replaced by N40 to provide two halide-coordinating residues in the reactive center of DmxA Q/N variant. Position of side-chain of N40 in the variant DmxA Q/N enables stronger binding of the halide released during the S_N_2 reaction. Structural analysis of DmxA reveals quite narrow tunnels, while the most important bottleneck in the main tunnel is formed by the residues M177 and F246. These residues were replaced by alanine in the variant DmxA MF/AA, resulting in a widely opened main tunnel. The white stars represent positions of the active sites in the enzyme structure, with respect to the tunnels and/or catalytic residues.

The role of the disulfide bridge in DmxA wt dimerization and stability was investigated by analyzing the C249S variant (designated here as DmxA C/S, Figure 4). Native gel electrophoresis confirmed that this variant exists as a pure monomer in the solution (Figure S3) and CD spectroscopy verified the correct folding and secondary structure of the variant (Figure S2). To independently verify the formation of a disulfide bridge in DmxA, both wt and DmxA C/S were subjected to proteolytic cleavage by pepsin and the resulting peptides were analyzed by MALDI-MS/MS. The formation of a disulfide bridge in DmxA was confirmed by detection of a signal at 3402.6 Da. This ion yielded MS/MS fragments characteristic of presence of disulfide dimer of WMDRCGLHHHHHH peptide (Figure S5). On the contrary, such signal was not detected in DmxA C/S mutant, proving the absence of disulfide bridge in the variant.

The determination of the melting temperature for the variant revealed that the cysteine bridge in DmxA wt dimer is not responsible for the unusual stability of the enzyme since DmxA C/S exhibited almost the same thermostability (*T*_m,app_ = 65.3 ± 0.2 °C, Figure 1) as the wild-type. Simultaneously, no significant changes between activity (Table S1) or enantioselectivity (Table S3) of DmxA C/S and DmxA wt were observed. PCA clustered DmxA C/S to the same SSG (SSG-I) as the wild-type (Figure 2). To exclude any potential effect of intersubunit disulfide bridge on the enzyme stability, we compared temperature profile (Figure S6) and the enzyme activity after repeated freeze–thaw cycles of DmxA wt and DmxA C/S (Figure S7). Both enzymes exhibited similar dependence of activity on increasing temperature with the highest activity at 55 °C. Simultaneously, both enzymes preserved 95–100% of its original activity after 10 repeated freeze–thaw cycles indicating that disulfide bond has no effect on cold stability of the enzyme. (Figure S7). In conclusion, formation of a cysteine bridge between individual subunits of DmxA wt dimer has no effect on the enzyme stability or functionality.

### 3.4. Analysis of Tunnel Network and Q40 in DmxA Wild-Type

To get a deeper insight into the structural features of DmxA wt, four independent MD simulations were run for each monomer identified in the crystal structure of the enzyme. All analyses were performed on the last 100 ns of the MD simulations. To further verify the validity of these simulations, much longer independent calculations were performed on the monomer (chain A as identified in the crystal structure of the enzyme) and the dimer. Both simulations yielded a three-state partition corresponding to different positioning of the first helix in the cap domain (α4). The first two states (aggregated equilibrium probability of 75% and 77% in the monomer and dimer, respectively) represent different cases for the closed conformation of the enzyme; the third state corresponds to its open conformation. All analyses were performed on 1000 random frames representative of the equilibrium of the different Markov states identified in the MD simulations. Q40 was systematically observed to be rotated to a non-stabilizing position and forming hydrogen bonds with oxygen from Y68 and/or L203 (Figure 3B) in all simulations.

The analysis of tunnel network revealed two predominant pathways leading from the active site to the surface. The topology of these paths corresponds to the p1 (main) tunnel and p2 (slot) tunnel, previously reported for other HLDs [74], referring to tunnels that serve mainly as access pathway for substrate and releasing pathway for product, respectively. Both the main and the slot tunnels were closed for water molecules for most of the MD simulations with an average bottleneck radius of 1.2 Å (main) or 1.1 Å (slot) and with an average opening for 1.4 Å probe of 30% (main) and 5% (slot), in the case of the monomer and of 11% (main) and 7% (slot) in the case of the dimer (Table S4). The analysis of tunnel bottlenecks, i.e., the narrowest part of the tunnel, in at least 80% of the simulations on average, revealed four important residues: T145, I173, M177, and F246 for main tunnel and M131, I135, L210, and L247 for slot tunnel (Table S5).

### 3.5. Construction of Variant DmxA Q/N with Substituted Halide-Stabilizing Residue

DmxA wt possesses a unique halide-stabilizing residue, Q40, instead of the conventional N typical of other HLD-II members. To assess the effect of the unique halide stabilizing residue on DmxA wt stability and functionality, Q40 in the enzyme active site was replaced by asparagine resulting in the variant DmxA Q/N (Figure 4). The constructed variant was correctly folded, as verified by CD spectroscopy, and its stability was approximately 2 °C lower (*T*_m,app_ = 63.9 ± 0.2 °C) than DmxA wt (Figure 1), which indicates that Q40 interacts with other residues in the active site and provides local stabilization for the enzyme. Moreover, the replacement of the glutamine by asparagine was accompanied by the significant activity and substrate specificity modifications of the enzyme. DmxA Q/N exhibited an increased activity towards 11 out of 30 tested substrates compared to DmxA wt (Figure 2A). The highest activity improvement of the variant was observed in the reaction with 1,3-dibromopropane and 1,3-diiodopropane. PCA clustered DmxA Q/N to SSG-IV (Figure 2B) owing to its enhanced preference towards terminally brominated and iodinated propanes and butanes [10]. In contrast, DmxA wt has been found to cluster into SSG-I. A similar shift in the substrate specificity class has been previously achieved by engineering of the second halide-binding site in DbeA, another member of HLD-II subfamily [75]. The catalytic efficiency of DmxA Q/N was tested towards the same substrates used for the kinetic characterization of DmxA wt. The variant exhibited a 12-times enhanced *k*_cat_, as well as a four-times increased *K*_m_ (Table S2) towards 1,3-dibromopropane, suggesting that the higher catalytic activity of the variant is owing to an improved catalytic rate when compared to DmxA wt. On the contrary, in the reaction with 1,2-dibromoethane and 4-bromobutyronitrile, DmxA Q/N exhibited three- and two-times lower *k*_cat_ accompanied by 1.5- and 2-times higher *K*_m_ than DmxA, respectively. Moreover, no substrate inhibition could be observed with this variant towards the tested substrates. Enantioselectivity of the variant remained comparable with that of the wild-type (Table S3). The measurement of enzymatic activity at different temperatures showed that DmxA Q/N exhibited a temperature profile almost identical to that of DmxA wt and DmxA C/S, achieving its highest activity at 55 °C (Figure S6), thus implying that Q40 residue is not responsible for DmxA wt stability.

### 3.6. Mutagenesis of Tunnel Bottlenecks and Construction of DmxA MF/AA Variant

To open the main tunnel, all four identified most frequently occurring bottleneck residues were mutated in silico to smaller amino acids (T145A, I173V, M177A and F246A) in DmxA wt structure (Table S5). The effect of two substitutions (M177A and F246A) was predicted as destabilizing while the effect of the other two (T145A and I173V) was predicted as neutral. The combination of destabilizing mutations M177A and F246A was predicted as an additive with total ΔΔG of 8.8 ± 1.2 kcal/mol. The destabilizing mutations M177A and F246A were introduced in silico into each monomer of DmxA wild-type. Four independent MD simulations were run for each mutated monomer. All analyses were performed on the last 100 ns of each MD simulation. The analysis of tunnel network revealed two major pathways leading from the active site to the surface which was in the correspondence with DmxA wild-type. The main tunnel was predominantly open for water molecules in MD simulations (average opening for 1.4 Å probe of 67%) with an average bottleneck radius of 1.5 Å. Thus, the opening was improved 2.2–5.5 times over the wild-type one. The slot tunnel was not affected by the mutations and remained closed for water molecules for almost all of the MD simulations (average opening for 1.4 Å probe of 5%) with an average bottleneck radius of 1.1 Å (Table S4).

To verify that the closed tunnel is the main reason of DmxA wt stability, a double-point mutant M177A/F246A, referred to here as DmxA MF/AA (Figure 4), was constructed and biochemically characterized. The constructed mutant exhibited 9 °C decrease in apparent melting temperature (*T*_m, app_ = 56.9 ± 0.3 °C; Figure 1) when compared with DmxA wt. This observation confirmed our hypothesis that the narrow tunnel is responsible for the unusual stability of DmxA enzyme. Such narrowness is probably induced by strengthened hydrophobic interactions due to the tight packing of the amino acids forming the walls of such closed access tunnel. Although the changes in the substrate specificity profile were not as prominent as in the case of DmxA Q/N, the DmxA MF/AA variant was clustered within SSG-IV (Figure 2) with a preference for brominated and iodinated substrates. The kinetic parameters of the constructed variant (Table S2) revealed that the introduced mutations hampered the substrate binding (represented by the increase of *K*_0.5_) and lowered the catalytic efficiency on the tested substrates when compared with DmxA wt. The enantioselectivity of DmxA MF/AA towards ethyl 2-bromopropionate compared to that of DmxA wt did not change (*E* > 200), but dropped significantly in the case of 2-bromopentane (*E* = 14), suggesting that either M177 or F246 is important for enantiodiscrimination of brominated alkanes by the enzyme (Table S3).

## 4. Discussion

The biochemical and structural analyses of a new haloalkane dehalogenase DmxA from Antarctic bacterium *Marinobacter* sp. ELB17 revealed the paradoxical stability of the enzyme. Despite its psychrophilic origin, DmxA wt exhibited the highest apparent melting temperature (*T*_m,app_ = 65.9 ± 0.1 °C) of all biochemically characterized HLD-II members, and the second highest of all thus far characterized wild-type HLDs. The only HLD exhibiting higher apparent melting temperature than DmxA was DhmeA from halophilic archaea *Haloferax mediterranei* ATCC 33500, which belongs to the subfamily HLD-III [76]. The enzyme DhmeA from subfamily HLD-III is a multimeric protein with *T*_m,app_ = 70.6 °C. The tertiary structure of DhmeA is not available at this moment, preventing its direct comparison with DmxA. Several thermostable enzymes from psychrophiles and mesophiles have been reported in the literature; however, their unexpected thermostability remains poorly understood [77]. Only two studies focused on such paradoxically thermostable enzymes from psychrophilic and mesophilic origin have attempted so far to explain the structural basis of their stability [25,26], suggesting that either the flexibility of a global structure [25] or the presence of extra disulfide and salt bridges [26] are responsible for the unusual thermal stability of these enzymes.

In this study, several structural features of DmxA wt were analyzed in detail to explain its extraordinary stability. DmxA wt was found to form a dimer via a cysteine bridge, which may be formed only after heterologous expression in *E. coli* and purification, while is less likely to occur in the cytoplasm of the original host organism. This is the first observation ever of HLD oligomerization mediated by the covalent bond. Thus far, the reported dimers of DbjA and DbeA were formed through the hydrophobic interactions of C-terminal α-helices [71,75,78]. Although cysteines in protein structures often serve as stabilizing elements, the S-S bridge between DmxA wt units in its dimeric form did not have any effect on the stability or the activity of the enzyme. Furthermore, our experiments revealed that the presence or absence of such cysteine bridge neither influenced any of the catalytic properties nor the storage stability of the enzyme. It is also worth mentioning that the DmxA wt oligomer equilibrium varies in solution, and only a small part of the enzyme (up to 30%) is present as a dimer. Therefore, the disulfide bridge does not contribute to the overall stability of the enzyme.

A unique composition of the catalytic pentad was observed both in DmxA wt sequence and crystal structure, containing a glutamine instead of the typical asparagine present in other members of the subfamily HLD-II [8]. Another HLD with an unusual halide-stabilizing residue (DatA containing tyrosine instead of tryptophan) was described earlier [9]. In this case, the unique halide stabilizing residue decreased enzyme stability (by 3.5 °C) and only moderately affected enzyme specificity [79]. The position of the Q40 side chain in DmxA wt structure disables its functioning as a halide-stabilizing residue. When replacing the glutamine with asparagine, the melting temperature of the enzyme decreased by 2 °C (*T*_m,app_ = 63.9 ± 0.2 °C) confirming a small stabilizing effect of glutamine interactions in the protein core. Moreover, an interesting shift in substrate specificity from SSG-I to SSG-IV was observed on the variant DmxA Q/N. Such changes in the substrate specificity in HLDs are normally only be achieved after a sophisticated protein engineering [78,80]. In our case, a single mutation was sufficient to shift the enzyme substrate specificity group.

A deeper analysis of DmxA wt structure revealed an unusually small hydrophobic pocket containing the enzyme active site connected with the surrounding solvent by very narrow and closed tunnels. The presence of phenylalanine and methionine as bottleneck residues was not overly surprising since they often provide stabilization of protein structures via hydrophobic interactions [15]. Replacement of bulky residues with alanines in the main tunnel opened the tunnel and suppressed stabilization of the tunnel structure via interactions provided by the bottleneck residues. This led to the destabilization of the protein core represented by 9 °C decrease of the enzyme apparent melting temperature. Engineering of the tunnels connecting buried enzyme active site with the surrounding solvent has previously been described as a powerful strategy for enzyme stabilization [81,82,83]. In this study, we proved that narrow tunnels play a crucial role in the paradoxical stability of DmxA due to multiple van der Waals and hydrophobic interactions, although they are not the only factor responsible for the unique properties of the enzyme.

To decipher the high stability of the new haloalkane dehalogenase DmxA wt, we investigated three unique features of the enzyme, corresponding to three possible sources of its stabilization. Both replacements of glutamine with asparagine and opening of the main tunnel brought desired outcomes in terms of decreased stability and improved activity. More importantly, introducing only two mutations, we managed to turn the thermostable enzyme into a mesophilic-like counterpart, with a melting temperature comparable to that of other related HLDs and a significantly improved catalytic performance. If we combined the effects of mutations in DmxA Q/N and DmxA MF/AA variants, the consequent enzyme would result in a highly active catalyst.

## Figures and Tables

**Table 1:** Diffraction data collection and refinement statistics.

Data Collection Statistics	
X-ray Source	ESRF Grenoble, ID29
Wavelength (Å)	0.972
Resolution range (Å)	100.0–1.45 (1.49–1.45)
Space group	*P*2_1_2_1_2_1_
Unit-cell parameters (Å; °)	a = 43.37, b = 78.34, c = 150.5; α = γ = β = 90.0
Total no. of measured intensities	484,657 (37,044)
Number of unique reflections	39,029 (5978)
Redundancy	5.28 (5.52)
Average I/σ(I)	8.02 (2.1)
Completeness (%)	99.7 (99.9)
R_meas_^a^ (%)	9.1 (71.9)
R_merge_^b^ (%)	11.2 (62.1)
Wilson B (Å^2^)	21.048
**Refinement**	
Resolution range(Å)	75.26–1.45 (1.48–1.45)
No. of reflections in working set	86,980 (6373)
No. of reflections in test set	4589 (329)
R value (%) ^c^	17.32 (28)
R_free_ value (%) ^d^	21.38 (30.5)
RMSD, bond lengths (Å)	0.0188
RMSD, angles (°)	1.9274
No. of atoms in AU	5475
No. of water molecules in AU	599
No. of acetate ions in AU	3
Mean B value (Å^2^)	18.62
Ramachandran plot statistics:	
Residues in preferred regions (%)	91.5
Residues in allowed regions (%)	3.76
Residues outliers (%)	1.08
PDB code	5MXP

Values in parentheses are for the highest-resolution shell. ^a^ R_meas_ = Σ*_hkl_*[*N*/(*N* − 1)]^1/2^ Σ_i_|*I*_i_(*hkl*) − 〈*I*(*hkl*)〉|/Σ*_hkl_*Σ*_i_I_i_*(*hkl*), where 〈*I*(*hkl*)〉 is the mean of the *N*(*hkl*) individual measurements *I_i_*(*hkl*) of the intensity of reflection *hkl*. ^b^ R_merge_ = S_hkl_ S_i_ |I_i_(hkl) − (I(hkl))|/S_hkl_ S_i_ I_i_(hkl), where *I_i_(hkl)* is the *i*th observation of reflection *hkl* and *<I(hkl)>* is the weighted average intensity for all observations of reflection *hkl*. ^c^ R-value = ||Fo| − |Fc||/|Fo|, where Fo and Fc are the observed and calculated structure factors, respectively. ^d^ R_free_ is equivalent to R value but is calculated for 5% of the reflections chosen at random and omitted from the refinement process [73].

## Supplementary Materials

The following are available online at https://www.mdpi.com/2076-2607/7/11/498/s1.
