# Supplementary material for: Deciphering the Structural Basis of High Thermostability of Dehalogenase from Psychrophilic Bacterium Marinobacter sp. ELB17
Source: Microorganisms. 2019 Oct 28;7(11):498. doi: 10.3390/microorganisms7110498 (PMC6920932; doi:10.3390/microorganisms7110498)
Supplement: Supplementary file 1 [file microorganisms-07-00498-s001.pdf]

SUPPLEMENTAL INFORMATION

Deciphering the structural basis of high thermostability of dehalogenase from psychrophilic bacterium from *Marinobacter* sp. ELB17

Running title: Paradoxically thermostable dehalogenase enzyme

Lukas Chrast[1], Katsiaryna Tratsiak[2,4], Joan Planas-Iglesias[1], Lukas Daniel[1], Tatyana Prudnikova[2], Jan Brezovsky[1,3], David Bednar,[1,3] Ivana Kuta Smatanova[2], Radka Chaloupkova[1,5], Jiri Damborsky[1,3]*

[1]Loschmidt Laboratories, Department of Experimental Biology and RECETOX, Faculty of Science, Masaryk University, Kamenice 5/A13, 625 00 Brno, Czech Republic

[2]Institute of Chemistry and Biochemistry, Faculty of Science, University of South Bohemia Ceske Budejovice and Institute of Microbiology Academy of Sciences of the Czech Republic, Branisovska 1760, 370 05 Ceske Budejovice, Czech Republic

[3]International Clinical Research Center, St. Anne's University Hospital, Pekarska 53, 656 91 Brno, Czech Republic

[4]Institute of Organic Chemistry and Biochemistry ASCR, v.v.i. Flemingovo nam. 2, 166 10 Prague 6, Czech Republic

[5]Enantis Ltd., Biotechnology Incubator INBIT, Brno, Czech Republic

*Corresponding author. Mailing address: Loschmidt Laboratories, Department of Experimental Biology and RECETOX, Faculty of Science, Masaryk University, Kamenice 5/A13, 625 00 Brno, Czech Republic. Phone: 420-5-4949 3567. Fax: 420-5-4949 6302. E-mail: jiri@chemi.muni.cz.

cat**ATG**ACCACCCAGAAACCGGCTGATTTTCCGTATCCTAGCCATTTTGCCGATGTGCTG
GGCTCCCGCATGCATTATGTTGAACATGGTAACGGTGATCCTCTGCTGTTTCTGCATGGC
CAGCCAACTTGGTCTTATCTGTGGCGTAAAGTTCTGCCTGAACTGGAAGGCAAAGGTCGT
CTGATCGCTGTTGATCTGATTGGTTATGGTATGAGTGATAAACCTGATATCCCTTATGAT
ATTGATGATCATATCCGCTATCTGGATGGCTTTATCGAAGCACTGGGCCTGGATCGTATT
ACAATCGTGTGTCATGATTGGGGCAGCTTTTTTGGTTTTCATTATGCCCATCGTCATCCG
GAACGTATTAAAGGTCTGGCTTTTATGGAAGCAATGCTGAACCCGATTCCGGGCTATGAT
GCCTTTGATCCTCAGACACGTGCCTTTTTTCAGACCCTGCGTAGCAGTCAGGCTAACGCC
GAACGTATGATGATGGATGAAAATCAGTTTGTGGAAAACATCCTGCCGGCAATGATTTGT
CGTCCGCTGGAACGCCAGGAACTGGATGCTTATCGTGCTCCATGGACCGATCGCCAGTCT
CGCCGTATCCTGTGTACATTTCCTCAGAACCTGTGTATTGGCAAAGAACCGGCAAGCGTG
TATCGTATGCAGACCGCCTATATCGAATGGCTGGGTCAGACAGATCTGCCAAAACTGCTG
ATTCATGCCGAACCTGGTTTTCTGATTCCGGCACCGGCTGTTGATCAGTATCGCCAGCAG
CTGCCTAATCTGGAAACCGCCTTTGTTGGTAGTGGCCTGCATTATATCCAGGAAGATCAG
CCACAGAAAATTGGTCAGGCCATTGCTCAGTGGATGGATCGTTGTGGCCTGCATCATCAT
CATCATCAT**TAA**aagctt

Figure S1. DNA sequence of codon-optimized *dmxA* gene for expression in *Escherichia coli*. Restriction sites *Nde*I and *Hind*III are highlighted in blue and yellow; START and STOP codons are highlighted in bold; C-terminal His-tag sequence is highlighted in green.

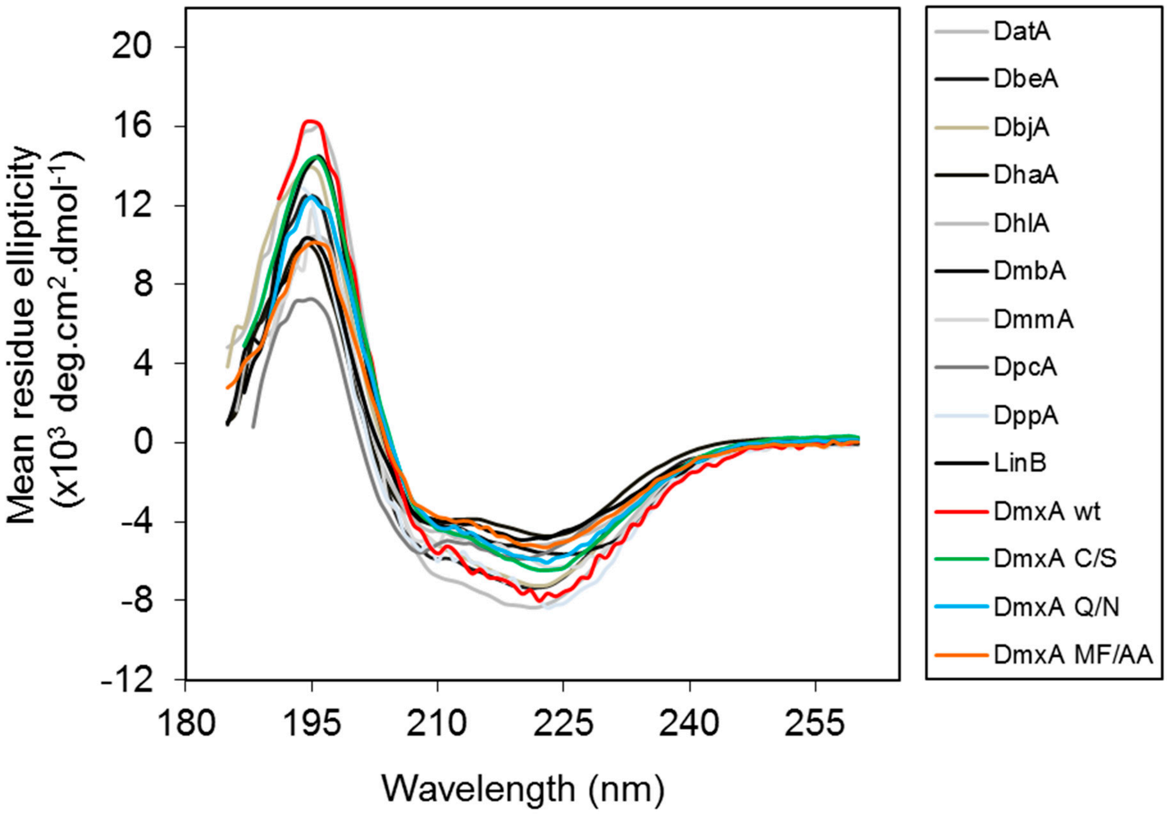

Figure S2. Circular dichroism spectra of selected haloalkane dehalogenases and DmxA variants.

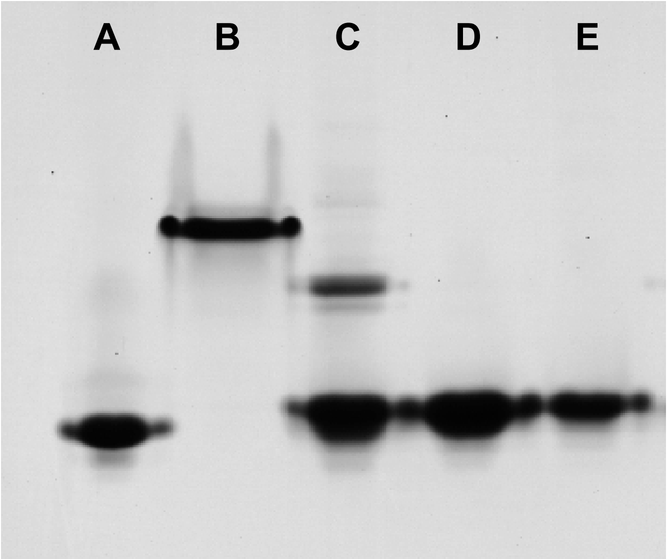

Figure S3. Native PAGE of selected haloalkane dehalogenases and DmxA variants: A – LinB (monomer), B – DbjA (dimer), C – DmxA (monomer + dimer), D – DmxA with 10 mM DTT (monomer), E – DmxA C/S (monomer)

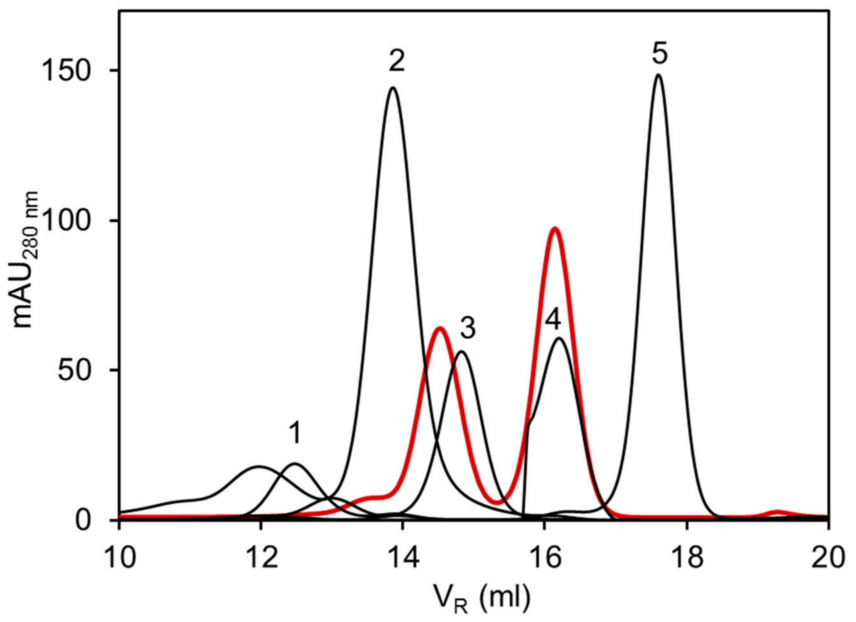

Figure S4. Size-exclusion chromatography of DmxA wt including standards. Red line – signal of native DmxA wt; 1 - Aldolase (158 kDa), 2 - Conalbumin (75 kDa), 3 - Ovalbumin (44 kDa), 5 - Carbonic anhydrase (29 kDa), 5- Ribonuclease A (13.7 kDa).

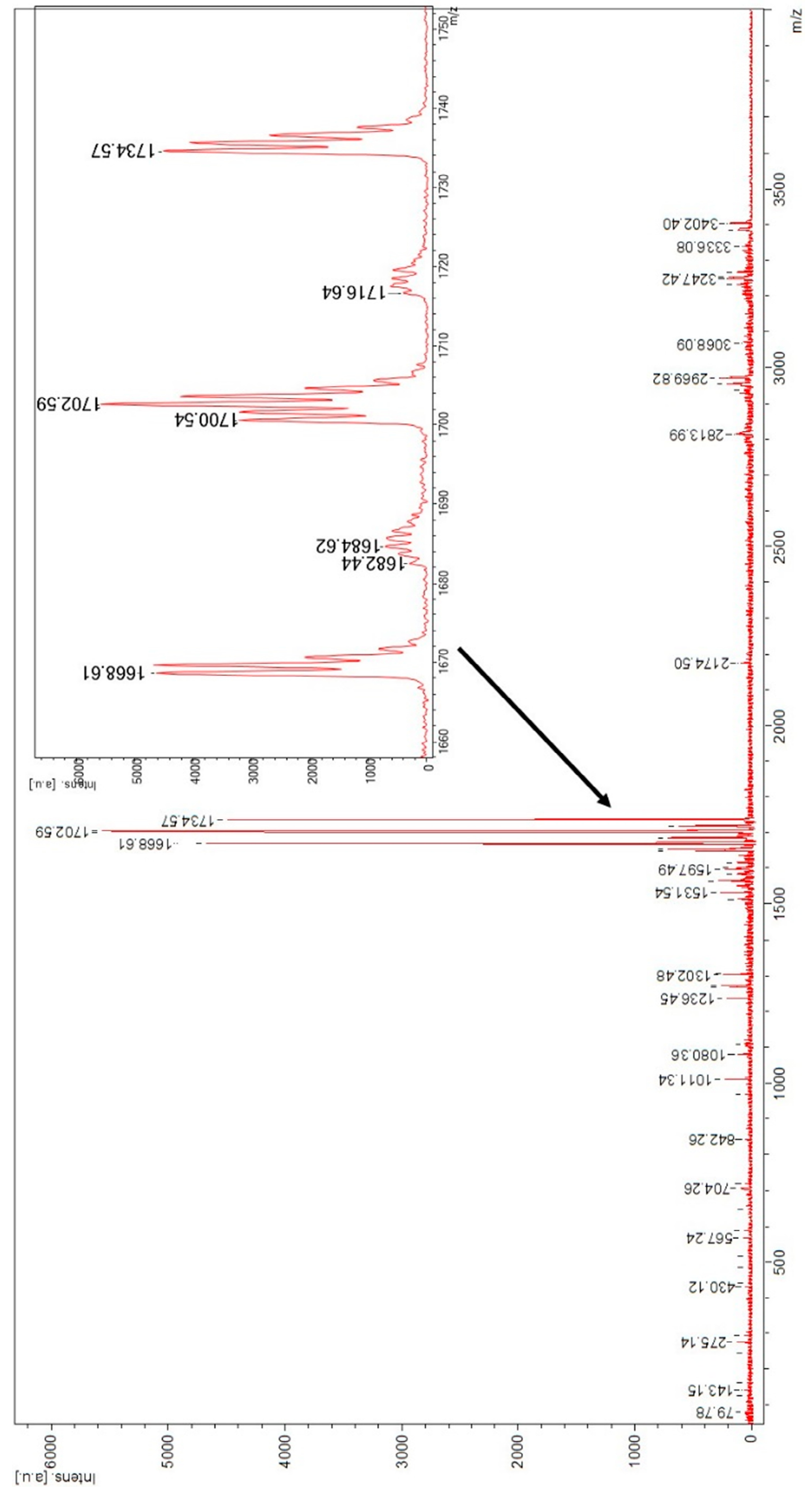

Figure S5. MALDI-MS/MS spectrum of DmxA after pepsin proteolysis. Black arrow indicates position of peptide 3402.6 (WMDRC*GLHHHHHH -S-S- dimer: MH+) which confirms presence of C-terminal disulphide bridge in the protein structure.

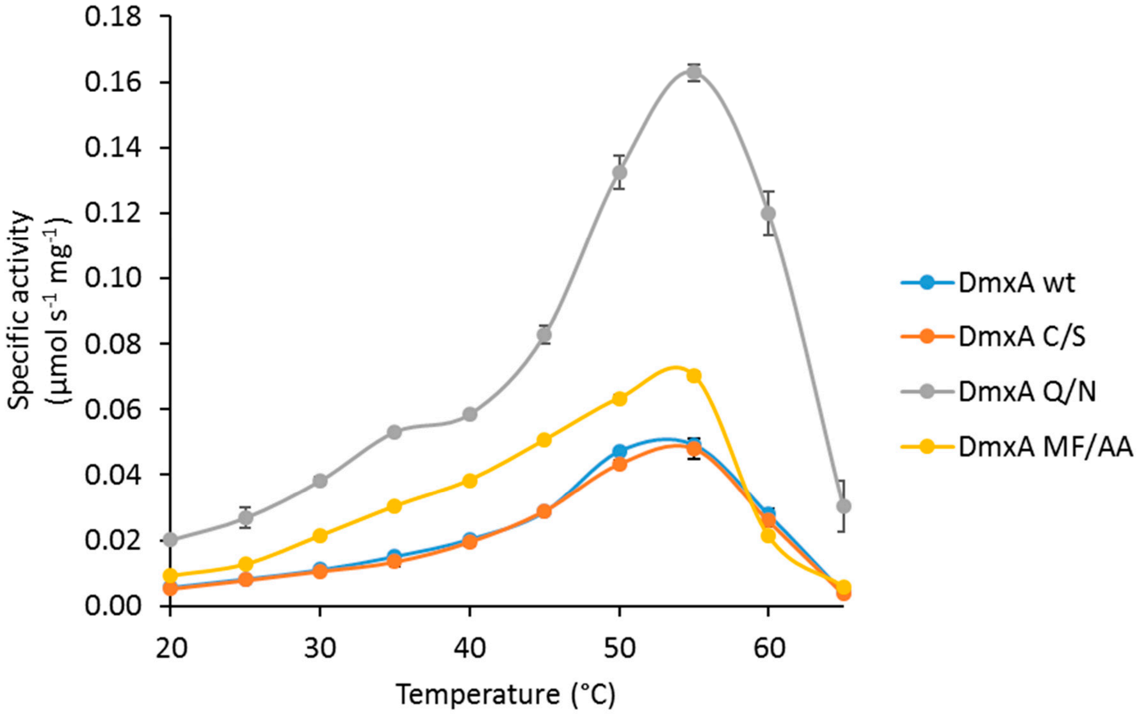

Figure S6. Temperature profiles of DmxA and its variants. Activity assays were performed in temperatures ranging from 20 °C to 65 °C, data were collected with 1,3-diiodopropane as substrate. Profiles depict specific activity in various temperatures, with maximum activity at 55 °C for all enzymes. Activity assays were performed in triplicates, error bars represent standard deviation.

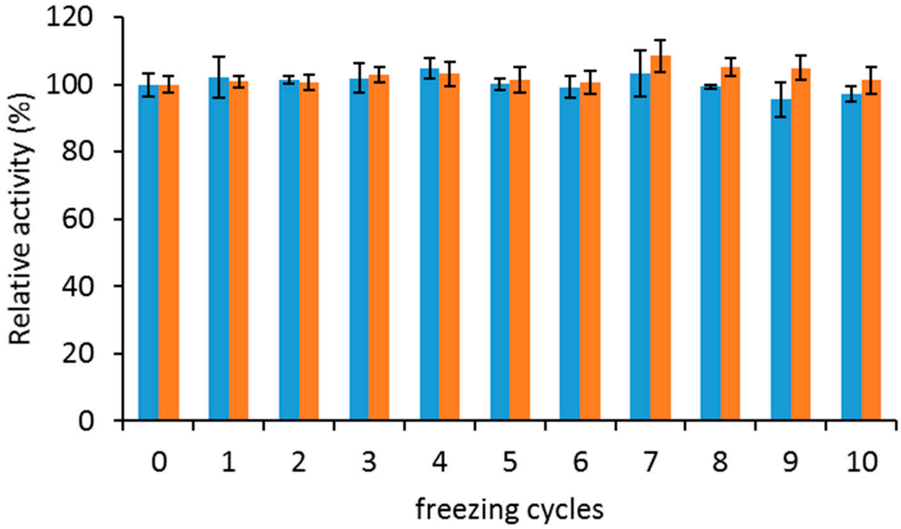

Figure S7. Freeze-thaw stability of DmxA wt (blue bars) and DmxA C/S variant (orange bars) monitored by activity testing before, during, and after 10 freeze-thaw cycles. No significant loss in activity of both enzyme variants after 10 cycles of freezing and thawing was observed confirming the stability and resistance to freezing damage of both DmxA variants. Experiments were performed in triplicates, bars represent mean values, error bars represent standard deviations.

Table S1. Specific activities[a] of DmxA and its variants determined towards a set of 30 different halogenated substrates.

Substrate		Specific activity (μmol s^{-1} mg^{-1})			
No.	Name	DmxA wt	DmxA C/S	DmxA Q/N	DmxA MF/AA
**4**	1-chlorobutane	0.005	0.002	0.001	0.001
**6**	1-chlorohexane	0.006	0.004	0.002	0.004
**18**	1-bromobutane	0.009	0.007	0.022	0.024
**20**	1-bromohexane	0.006	0.005	0.014	0.010
**28**	1-iodopropane	0.009	0.009	0.024	0.017
**29**	1-iodobutane	0.004	0.004	0.021	0.014
**31**	1-iodohexane	0.005	0.005	0.019	0.006
**37**	1,2-dichloroethane	ND	ND	ND	ND
**38**	1,3-dichloropropane	0.002	0.001	0.001	0.000
**40**	1,5-dichloropentane	0.005	0.003	0.004	0.003
**47**	1,2-dibromoethane	0.011	0.016	0.016	0.009
**48**	1,3-dibromopropane	0.030	0.035	0.084	0.055
**52**	1-bromo-3-chloropropane	0.024	0.024	0.041	0.040
**54**	1,3-diiodopropane	0.017	0.019	0.064	0.033
**64**	2-iodobutane	0.008	0.010	0.014	0.014
**67**	1,2-dichloropropane	ND	ND	ND	ND
**72**	1,2-dibromopropane	0.007	0.009	0.002	0.005
**76**	2-bromo-1-chloropropane	0.006	0.005	0.002	0.005
**80**	1,2,3-trichloropropane	0.000	0.000	0.000	0.000
**111**	bis(2-chloroethyl)ether	ND	ND	ND	ND
**115**	chlorocyclohexane	ND	ND	ND	ND
**117**	bromocyclohexane	0.005	0.007	0.004	0.006
**119**	(1-bromomethyl)cyclohexane	0.004	0.002	0.009	0.003
**137**	1-bromo-2-chloroethane	0.013	0.016	0.007	0.006
**138**	chlorocyclopentane	0.006	0.004	0.002	0.003
**141**	4-bromobutyronitrile	0.027	0.025	0.032	0.052
**154**	1,2,3-tribromopropane	0.019	0.022	0.008	0.006
**155**	1,2-dibromo-3-chloropropane	0.012	0.011	0.003	0.006
**209**	3-chloro-2-methylpropene	0.009	0.016	0.004	0.013
**225**	2,3-dichloropropene	0.004	0.002	0.001	0.002

[a]Each activity was measured in at least three independent replicates with standard deviations of less than 10%; ND – activity was not detected under used conditions.

Table S2. Steady-state kinetic parameters of DmxA and its variants.

Substrate	Variant	$K_{0.5}$ (mM)	k_{cat} (s^{-1})	n[a]	K_{SI} (mM)	b[b]	$k_{cat}/K_{0.5}$ (s^{-1} mM^{-1})
1,2-dibromoethane	DmxA wt	0.80	1.89	-[c]	1.66	-[c]	2.36
	DmxA Q/N	1.19	0.56	1.25	-[c]	-[c]	0.47
	DmxA MF/AA	1.34	0.43	1.52	18.61	-[c]	0.32
1,3-dibromopropane	DmxA wt	0.03	2.61	1.77	3.24	0.29	88.09
	DmxA Q/N	0.13	30.91	-[c]	6.68	-[c]	244.12
	DmxA MF/AA	0.13	1.91	2.29	-[c]	-[c]	14.76
4-bromobutyronitrile	DmxA wt	0.67	1.89	-[c]	5.72	-[c]	2.83
	DmxA Q/N	1.25	0.93	1.35	-[c]	-[c]	0.75
	DmxA MF/AA	0.93	1.25	1.53	-[c]	-[c]	1.35

[a]Hill index of cooperativity; [b]Factor of hyperbolic inhibition; [c]Not applicable; Standard deviations from at least three independent experiments with DmxA or its variants were within 10 %.

Table S3. Enantioselectivity of DmxA and its variants and other haloalkane dehalogenases.

Substrate	*E*-value							
	DmxA wt	DmxA C/S	DmxA Q/N	DmxA MF/AA	DatA[1]	DhaA[2]	LinB[2]	DbjA[2,3]
2-bromopentane	100	106	104	14	> 200	7	16	132
ethyl 2-bromopropionate	> 200	> 200	> 200	> 200	> 200	85	97	> 200

n.a. – not analysed; [1]Hasan et al. (2011); [2]Prokop et al. (2010); [3]Chaloupkova et al. (2011); Standard deviations from at least three independent experiments with DmxA or its variants were within 5 %.

Table S4. Characteristics of the top-ranked tunnel clusters in DmxA MF/AA variant using the probe radius 0.8 Å.

	MF/AA		WT monomer		WT dimer	
	main tunnel	slot tunnel	main tunnel	slot tunnel	main tunnel	slot tunnel
Average bottleneck radius [Å]	1.5 ± 0.1	1.1 ± 0.0	1.2 ± 0.3	1.1 ± 0.2	1.1 ± 0.3	1.1 ± 0.2
Average opening for 1.4 Å probe [%]	67 ± 23	5 ± 2	30 ± 6	5 ± 2	11 ± 6	7 ± 3

Table S5. List of residues forming the tunnel bottleneck in at least 80% of molecular dynamics simulations. The mutability scores were calculated by HotSpot Wizard[4].

Main tunnel			
Residue	Average percentage of formed bottleneck during MD [%]		Mutability score
	Monomer	Dimer	
T145	92.3 ± 4.6	98.1 ± 3.0	8
I173	85.2 ± 6.7	93.2 ± 8.3	6
M177	98.7 ± 1.1	–	6
F246	90.2 ± 3.8	–	7

Slot tunnel			
Residue	Average percentage of formed bottleneck during MD [%]		Mutability score
	Monomer	Dimer	
M131	98.5 ± 1.4	98.0 ± 1.6	6
I135	98.2 ± 1.4	88.2 ± 7.3	7
L210	82.4 ± 5.6	99.1 ± 1.4	5
L247	99.1 ± 0.9	–	5

[4]Sumbalova et al. (2018)

Table S6. Primers used for mutagenesis of DmxA*.

Mutagenic primers	
DmxA_C/S_Fw	5'-gtggatggatcgttctggcctgcatcatc-3'
DmxA_C/S_Rv	5'-gatgatgcaggccagaacgatccatccac-3'
DmxA_Q/N_Fw	5'-tctgctgtttctgcatggcaatccaacttggtcttatctgt-3'
DmxA_Q/N_Rv	5'-acagataagaccaagttggattgccatgcagaaacagcaga-3'
DmxA_MF/AA_Fw	5'-ttgtggaaaacatcctgccggcagcgatttgtcgtccgctggaac-3'
DmxA_MF/AA_Rv	5'-aacagccggtgccggaatcagagcaccaggttcggcatgaatc-3'
**Non-mutagenic primers**	
pET_Fw	5'-taatacgactcactataggg-3'
pET_Rv	5'-gctagttattgctcagcgg-3'

*Triplets for substitutions are highlighted in yellow.

References

1. Chaloupkova R, Prokop Z, Sato Y, Nagata Y & Damborsky J (2011) Stereoselectivity and conformational stability of haloalkane dehalogenase DbjA from *Bradyrhizobium japonicum* USDA110: the effect of pH and temperature. *FEBS J* 278, 2728-2738.

2. Hasan K, Fortova A, Koudelakova T, Chaloupkova R, Ishitsuka M, Nagata Y, Damborsky J & Prokop Z (2011) Biochemical characteristics of the novel haloalkane dehalogenase DatA, isolated from the plant pathogen *Agrobacterium tumefaciens* C58. *Appl Environ Microb* 77, 1881-1884.

3. Prokop Z, Sato Y, Brezovsky J, Mozga T, Chaloupkova R, Koudelakova T, Jerabek P, Stepankova V, Natsume R, van Leeuwen JGE, Janssen DB, Florian J, Nagata Y, Senda T & Damborsky J (2010) Enantioselectivity of haloalkane dehalogenases and its modulation by surface loop engineering. *Angew Chem Int Edit* 49, 6111-6115.

4. Sumbalova, L., Stourac, J., Martinek, T., Bednar, D., Damborsky, J. (2018) HotSpot Wizard 3.0: Web server for automated design of mutations and smart libraries based on sequence input information. *Nucl Acids Res* 46, W356-W362.
